# *FTO* Variant rs1421085 Associates With Increased Body Weight, Soft Lean Mass, and Total Body Water Through Interaction With Ghrelin and Apolipoproteins in Arab Population

**DOI:** 10.3389/fgene.2019.01411

**Published:** 2020-01-31

**Authors:** Prashantha Hebbar, Mohamed Abu-Farha, Anwar Mohammad, Fadi Alkayal, Motasem Melhem, Jehad Abubaker, Fahd Al-Mulla, Thangavel Alphonse Thanaraj

**Affiliations:** Research Division, Dasman Diabetes Institute, Dasman, Kuwait

**Keywords:** biomarkers, *FTO*, ghrelin, obesity, soft lean mass, total body water

## Abstract

Association studies have implicated single nucleotide polymorphisms (SNPs), particularly rs1421085, from the fat mass and obesity-associated (*FTO*) gene with body composition phenotypes, obesity, dietary intake, and physical activity in European, East Asian, and African populations. However, the impact of the rs1421085 variant has not been sufficiently tested in ethnic populations (such as Arabs) with high levels of obesity. Further, there is a lack of studies identifying biomarkers that interact with *FTO*. Therefore, we investigated the association of rs1421085 with obesity and body composition traits and metabolic biomarkers in Arab population. We genotyped rs1421085 SNP in 278 Arab individuals, where multiple biomarkers relating to obesity, inflammation, and other metabolic pathways were quantified. We performed genetic association tests under additive mode of inheritance using linear regression models and found association of rs1421085_C allele with higher levels of body weight, soft lean mass (SLM), and total body water. Examination (using linear regression models under dominant mode of inheritance) of correlation among biomarkers and interaction with genotypes at the variant revealed that measures of these three body composition traits were found mediated by interaction between carrier genotypes (TC+CC) and measures of ghrelin, ApoA1, and ApoB48. Lean body mass (LBM), to which SLM contributes, is an important determinant of physical strength and is a focal point in studies on sarcopenia. Low LBM is known to be associated with higher risk of cardiometabolic disorders. Thus, the finding on the *FTO* variant as a genetic determinant of SLM *via* interaction with ghrelin, ApoA1, and ApoB48 is important.

## Introduction

The fat mass and obesity-associated (*FTO*) gene is a well-established obesity-susceptibility locus. Early genome-wide association studies (GWAS) associated *FTO* genetic variants with obesity traits in Europeans (Dina et al., 2007; Frayling et al., 2007); subsequently, many of these risk variants were replicated in Africans, South and East Asians, and other populations. Furthermore, association of *FTO* variants with type 2 diabetes mellitus (T2DM), independent of body mass index (BMI), has been demonstrated in East and South Asians (Li et al., 2012) and Scandinavians (Hertel et al., 2011). It is also the case that *FTO* is associated with body composition traits such as lean body mass, fat free mass, and body fat percentage in people of European ancestry (Zillikens et al., 2017; Hubel et al., 2019).

*FTO* has been functionally implicated in both adipogenesis and tumorigenesis and on the underlying m^6^A-dependent demethylase activity which influences several mRNA processing events (Gerken et al., 2007; Bartosovic et al., 2017; Deng et al., 2018). The most notable genetic variants from *FTO* associated with obesity and body composition traits are a cluster of at least 20 common SNPs from its first intron (Loos and Yeo, 2014). Despite the association of many *FTO* genetic variants with obesity traits in humans, elucidation of the mechanistic basis for these associations has been elusive. Obesity has reached epidemic proportions, and understanding its genetic basis will help mitigate obesity-associated illness. In this context, there is an urgent need to decipher the causal mechanism for the role of *FTO* noncoding variants in the aetiology of obesity and its related disorders (Wu and Arora, 2016).

Among the published *FTO* variants, rs1421085_T > C is of particular interest; previously published global GWAS [as listed in NHGRI-EBI GWAS Catalog (Buniello et al., 2019)] associated the rs1421085 variant with risk for obesity (European) (Wheeler et al., 2013), increased BMI (European) (Felix et al., 2016), risk for T2DM (Japanese, European) (Imamura et al., 2016; Kichaev et al., 2019), increased dietary macronutrient intake (European) (Tanaka et al., 2013), increased body fat percentage (European), decreased hand grip strength (British) (Tikkanen et al., 2018), increased fat-free mass (European) (Hubel et al., 2019), decreased pulse pressure (East Asian) (Takeuchi et al., 2018), and preference for morning sleep (European) (Kichaev et al., 2019). A recent study by Claussnitzer et al. (2015) ascribed a critical role for the rs1421085 variant, in adipocyte-thermogenesis regulation pathway through interaction with *ARID5B*, *IRX3,* and *IRX5 genes, by* utilising an integrative approach combining data on the regulatory role of the variant on its upstream and downstream gene regulators that were identified from genetic and epigenetic profiles of animal models. It therefore indicates that manipulation of this pathway may regulate obesity levels. Similar approaches are needed to delineate the influence of genetic variants on the aetiology of obesity, and this may be achieved through deep phenotyping of biomarkers, known to be involved in related biological processes, and by studying these genetic variants in various cohorts (Price et al., 2015; Szendroedi et al., 2016).

According to the most recent World Health Organization report, worldwide prevalence of obesity has tripled since 1975 (https://www.who.int/news-room/fact-sheets/detail/obesity-and-overweight). The prevalence of obesity is most noticeable in the Gulf region, where nine countries ranked highest in the obesity statistics among adults aged ≥18 years. Despite its high prevalence in the Gulf region, the Arab population is under-represented in global studies of obesity. In this study, we evaluated the influence of the *FTO* rs1421085 variant on various anthropometric and body composition measures and obesity, inflammatory, and cardiovascular biomarkers in a cohort of Kuwait Arab subjects.

## Materials and Methods

### Study Participants

The study protocol was reviewed and approved by the Ethical Review Committee of Dasman Diabetes Institute and was conducted according to the guidelines of the Declaration of Helsinki and of the US Federal Policy for the Protection of Human Subjects. Participants were Kuwaitis of Arab ethnicity, which was confirmed through detailed questioning on parental lineage up to three generations. The study population consisted of 278 subjects whose data on age, sex, comorbidities (e.g., diabetes and cardiovascular complications) and baseline characteristics, and vital signs such as height, weight, and waist circumference (WC) were recorded during enrolment. Details on medications taken by the participants for lowering lipid levels and diabetes were collected and used in correction procedures with the association statistics. All participants signed the consent form before participating in the study.

### Sample Collection and Processing

Collection of blood samples and measurement of vital signs were performed in accordance with established institutional guidelines. DNA was extracted using the Gentra Puregene^®^ kit (Qiagen, Valencia, CA, USA). Quant-iT™ PicoGreen^®^ dsDNA Assay Kit (Life Technologies, Grand Island, NY, USA) and Epoch Microplate Spectrophotometer (BioTek Instruments) were used to quantify DNA; absorbance values at 260–280 nm were checked for adherence to an optical density range of 1.8–2.1.

### Estimation of Plasma Levels of Various Biomarkers

The plasma levels of 46 biomarkers listed in **Supplementary Table S1** were determined. Briefly, blood samples were collected in EDTA-treated tubes. Plasma, separated by centrifugation, was aliquoted and stored at −80°C. The levels of apolipoproteins, except for ApoB48, were determined using Luminex assay from Millipore. The levels of inflammatory markers were determined using Luminex immunobead array (R&D Systems). The data were processed using the Bio-Plex Manager Software version 6 (Bio-Rad) and analysed using five-parametric curve fitting. High Sensitivity C-Reactive Protein (HsCRP) level was measured by enzyme-linked immunosorbent assay, as reported earlier (Abu-Farha et al., 2014; Abu-Farha et al., 2016) and adipokines were measured using Luminex assay (R&D Systems). Analytes provided by the manufacturer were used as quality controls to validate specificity and sensitivity.

### Bioelectric Impedance Measurements

Body composition of the participants was measured using Bio-impedancemetry Body Composition Analyzer IOI 353 (Jawon Medical Co., South Korea). Total body water (TBW) is the summation of body water that exists inside the cell membrane (intracellular) and water outside the cell membrane (i.e., blood and intestinal fluid, extracellular). Soft lean mass (SLM) is the addition of TBW and proteins in the body and is made up of skeletal and smooth muscle (Bera, 2014). Lean body mass (LBM) is the summation of SLM and minerals. Percentage body fat (PBF) was estimated by subtracting LBM from the total body weight.

### Targeted Genotyping

The TaqMan^®^ Genotyping Assay on ABI 7500 Real-Time PCR System from Applied Biosystems (Foster City, CA, USA) was used to perform candidate SNP genotyping. Next, 10 ng of DNA, 5× FIREPol^®^ Master Mix (Solis BioDyne, Estonia), and 1 µl of 20× TaqMan^®^ SNP Genotyping Assay constituted each polymerase chain reaction sample, and thermal cycling conditions were set at 60°C for 1 min and 95°C for 15 min followed by 40 cycles of 95°C for 15 s and 60°C for 1 min. Sanger sequencing, using the BigDye™ Terminator v3.1 Cycle Sequencing on an Applied Biosystems 3730xl DNA Analyzer, was performed for selected cases of homozygotes and heterozygotes to validate genotypes determined by the above techniques.

### Power Calculation

We used the Quanto software tool (University of Southern California, Los Angeles, CA, USA) to calculate the power of the study cohort and its ability to delineate quantitative trait variability at a given power (which we set at 80%). We considered the underlying genetic model to be based on additive mode of inheritance; we performed the power calculation considering “gene only” contributions with the underlying genetic model as based on additive mode of inheritance (AA versus AB versus BB) or under “gene-environment (GxE)” contributions (Parnell et al., 2014) with the underlying genetic model as based on dominant mode of inheritance (AA+AB versus BB, where A is the effect allele; see (Zhao et al., 2016) for definitions of genetic models for complex disorders). We set a P-value threshold of ≤ 0.05 for considering a type 1 error as significant. Genetic effect that accounts for at least 0.1%–5% variance in the trait was detected by letting the R_G_^2^ (estimate for marginal genetic effect) to assume values in the range of 0.001–0.05 in step of 0.005. We considered the population (mean ± standard deviation) of the quantitative trait and of the environment term (interaction trait) in these calculations.

### Quality Assessment of SNP and Trait Measurements and Statistical Association Analysis

We used PLINK (version1.9) to assess quality and statistical association of the rs1421085 SNP. We calculated minor allele frequency (MAF) and Hardy–Weinberg equilibrium for the *FTO* variant. We considered any quantitative trait values < Q1- 1.5 * IQR or any value > Q3+1.5 * IQR as an outlier and excluded it from further statistical analysis.

A total of 60 traits and biomarkers (**Supplementary Table S1**) were considered in this study; it is to be noted that there can be dependency among some of these traits. Independent traits were identified from the summary statistics of Pearson correlation analysis among the 60 traits using the matSpD tool (Li and Ji, 2005) (available at https://sites.google.com/site/qutsgel/software/matspd-local-version). The analysis pointed to 23 independent traits.

We performed allele-based statistical association tests for rs1421085 with all 60 traits using linear regression adjusting for regular corrections toward age and sex. We also adjusted for diabetes medication and lipid-lowering medication. Correction for multiple testing was assessed by adjusting the P-value threshold for the 23 independent traits (0.05/effective number of independent traits), which was (0.05/23 = 0.0021). We also examined empirical P-values (P*_emp_*-value), generated using the max(T) permutation procedure available in PLINK, based on 10,000 permutations.

Further, correlations between the rs1421085 variant and levels of biomarkers/traits (such as SLM, TBW and weight), corrected for age/sex, as well as interactions of such correlations with other traits (example: SLM ~ genotypes+Age+sex+ApoA1*ApoB48) were evaluated using multivariable linear regression tests. Summary statistics on the relationships among the traits/biomarkers included percentage variation in response variable (r2), estimates denoting standardised beta-coefficients, standard error, and significance of test (P) for the genotype distributions. P-value threshold was set at 0.05.

## Results

### Sample Set for Each of the Traits

The quality control procedures for checking outliers in trait measurements and missing information on traits led to varying number of samples for different traits, with the maximum size of 270 for BMI.

### Characteristics of the Study Cohort

The summary statistics on the considered 60 traits and biomarkers of the 278 participants are summarised in **Supplementary Table S1**. The mean age of the cohort was 46.26 ± 12.39 years, with the ratio of males to females as 1:1.2. The cohort comprised mostly class I obese subjects with a mean BMI of 29.93 ± 5.18 kg/m^2^ and mean WC (99.36 ± 13.36 cm); 48% of the subjects were obese and 41% were diabetic. Mean HbA1c (6.31 ± 1.3%), low-density lipoprotein (3.13 ± 0.97 mmol/L), high-density lipoprotein (HDL) (1.2 ± 0.32 mmol/L), total cholesterol (5.02 ± 1.09 mmol/L), and triglyceride (1.22 ± 0.6 mmol/L) were normal or near optimal. Of the 278 subjects, 104 were taking medication for diabetes and 91 for lowering lipids.

After partitioning based on the genotypes (TT vs TC+CC) at the *FTO* rs1421085 variant, the cohort exhibited significant differences (P-value ≤ 0.05) in the mean values for weight, BMI, SLM, TBW, leptin, eotaxin, TNFα, MMP-9, MIP1α, IFNγ, and several interleukins (see **Supplementary Table S1**). The significances of differences were particularly strong for weight (P-value = 0.0047), IL-5 (P-value = 0.00076), IL-6 (P-value = 0.0046), IL-7 (P-value = 0.0043), IFNγ (P-value = 0.0078), and TNFα (P-value = 0.0052). Upon performing the matSpD analysis on these interleukins that showed significant differences, it was found that the effective number of independent variables were 3, suggesting that they were partially correlated.

### Association Between *FTO* rs1421085 Variant and Increased SLM, TBW, and Weight

The summary statistics of associations observed between the *FTO* variant and the phenotype traits, derived from allele-based association tests based on additive models corrected for age and sex, are listed in **Table 1** and **Supplementary Table S2**. Statistically significant associations (P-value threshold corrected for multiple testing ≤ 0.002) between the variant (with the minor allele C as the effect allele) and three traits (SLM, TBW, and weight) were observed; and values of P*_emp_*-value ≤ 0.05 were observed for these three association signals when the tests were further corrected for medication (**Table 1**). Though association signals for five other traits were at borderline P-values (C-peptide at P-value = 0.004, IFNγ at P-value = 0.005, eotaxin at P-value = 0.007, TNFα at P-value = 0.007, and IL-5 at P-value = 0.008), P*_emp_*-value did not reach the desired value of ≤ 0.05 even after the tests were corrected for medication (see **Supplementary Table S2**).

**Table 1:** Significant results (P-value < 0.002) of association tests for the *FTO* variant rs1421085 (with C as the effect allele) with phenotype traits, using genetic model based on additive mode of inheritance (TT versus TC versus CC).

Trait	Adjustments	Effect size (Beta value)	P-value	P*_emp_*-value
SLM	R	2.249	0.00103	0.055
	R+DM	2.402	0.00030	0.017
	R+LLM	2.265	0.00066	0.033
TBW	R	1.796	0.00103	0.055
	R+DM	1.920	0.00029	0.016
	R+LLM	1.808	0.00066	0.037
Weight	R	3.953	0.00221	0.114
	R+DM	4.566	0.00030	0.018
	R+LLM	4.374	0.00054	0.031

Association tests were adjusted for age, sex (R), and further confounders such as diabetes medication (DM) and lipid lowering medication (LLM). Results of association tests with other traits showing a P-value of ≤ 0.05 are given in **Supplementary Table S2**.

### Differences in the Mean Values of Weight, SLM, and TBW as per Genotype Distribution at the *FTO* Variant

**Figure 1:**
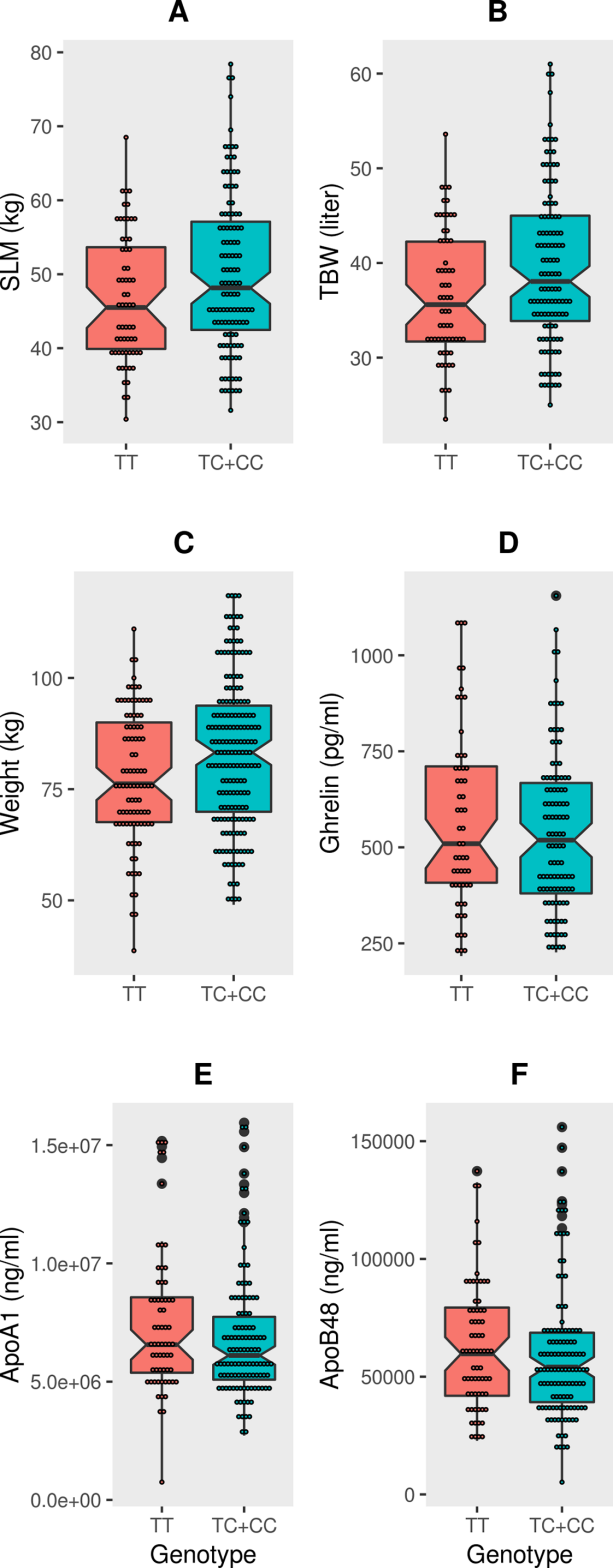
Box-plots displaying data distribution for the six phenotype traits such as **(A)** SLM, **(B)** TBW, **(C)** Weight, **(D)** Ghrelin, **(E)** ApoA1, **(F)** ApoB48 in individuals with genotypes (TC+CC) containing the effect allele or genotypes (TT) homozygous for reference allele at FTO variant rs1421085.

The mean values of the three traits (weight, SLM and TBW) associated with the *FTO* variant showed significant differences (P-value = 0.0047, P-value = 0.019 and P-value = 0.016, respectively) when the cohort was split as per the genotype distributions (TC+CC vs TT) (**Figure 1** and **Supplementary Table S1**). Upon grouping the cohort into non-obese and obese categories, amongs the three traits, only weight in the “obese” category retained statistical significance (P-value = 0.030) between the genotype distributions of (TC+CC) and (TT) (**Supplementary Table S3**). Thus, the impact due to the effect allele at the *FTO* variant on SLM, TBW, and weight was likely not due to obese subjects in the cohort. To confirm this, we performed linear regression analysis between each of these three traits and the genotype (TC+CC) with an interaction term involving “obesity status” or “diabetes status” (while correcting for age and sex) and examined the P-values (**Supplementary Table S4**). The P-values obtained were non-significant, and hence it may be considered that the association signal observed with SLM, TBW, and weight was not due to obesity or diabetes status of the subjects but was entirely due to the genotype of TC+CC, as opposed to TT.

### Do Other Traits and Biomarkers Interact With SLM, TBW, and Weight?

**Figure 2:**
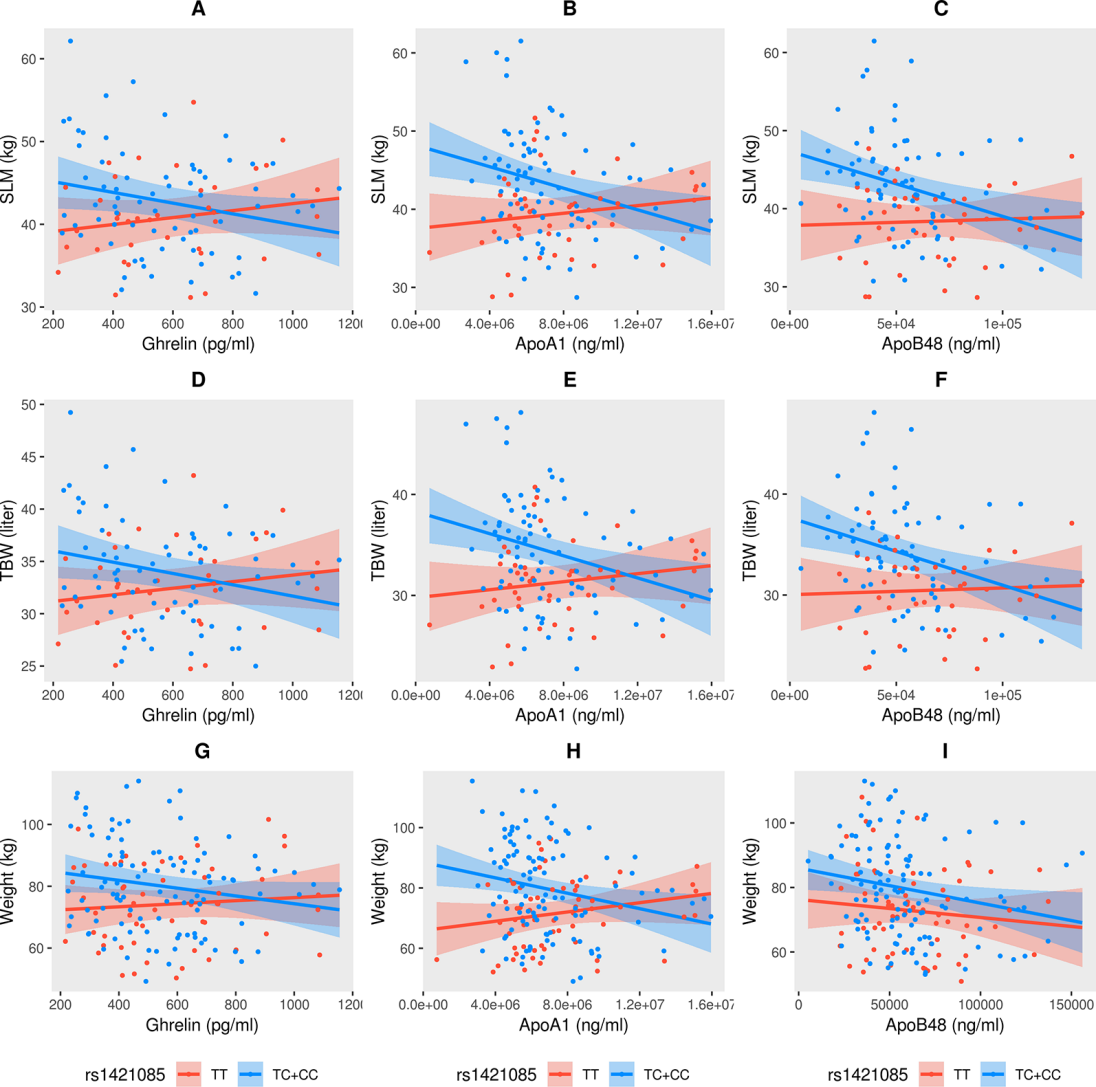
Significant interactions (negative correlation) observed in soft lean mass (SLM) **(A–C)**, total body water (TBW) **(D–F)**, and weight **(G–I)** with ghrelin, ApoA1, and ApoB48 with respect to the TC+CC genotype at *FTO* variant rs1421085.

Having noted that obesity or diabetes status of the participants does not have an effect on the association signal between SLM, TBW, weight, and the genotypes of TC+CC at the *FTO* variant, we analyzed the remaining traits and biomarkers of the 60 that we began with using the linear regression model. We found that with TC+CC genotypes (regressed against reference TT genotype), the P-values for interactions of SLM and TBW with ghrelin, ApoA1, and ApoB48 were significant, but for weight, only an interaction with ApoA1 was significant (**Table 2**). These results illustrate that for TC+CC genotypes at the *FTO* variant, levels of SLM, TBW, and weight increased and correspondingly levels of ghrelin, ApoA1, and ApoB48 decreased (**Figure 2**). Though the levels of body weight, total body water, and soft lean mass were seen correlated (at R^2^ > 0.20) with non-anthropometric traits such as TGL, HDL, FPG, HbA1c, insulin, PBF(%), leptin, ApoC3, glucagon, adiponectin, adipsin, ApoB48, and ghrelin (**Supplementary Table S5**) in study cohort, the *FTO* variant with TC+CC genotypes showed interactions only with ghrelin, ApoA1, and ApoB48” in linear regression adjusted for age, sex, and interacting trait.

**Table 2:** Linear regression model illustrating the link between the *FTO* variant rs1421085 and interaction between soft lean mass (SLM), total body water (TBW), and weight with ghrelin, ApoA1, and ApoB48.

Trait (response/dependent variable)	Genotype and/or interacting trait (Predict variable)	Estimate	Std. Error	P-value	Adjusted R-square (model)
SLM (TT count: 62; TC+CC count: 116)	TC+CC	8.161	3.259	0.013	0.587
	TC+CC*ghrelin	-0.010	0.005	0.041	
SLM	TC+CC	10.68	2.912	0.00036	0.663
	TC+CC*ApoA1	-9.34E-07	3.56E-07	0.0098	
SLM	TC+CC	9.51	2.85	0.0016	0.687
	TC+CC*ApoB48	-9.15E-05	4.27E-05	0.034	
TBW (TT count: 62; TC+CC count: 116)	TC+CC	6.533	2.61	0.013	0.562
	TC+CC*ghrelin	-0.0085	0.004	0.042	
TBW	TC+CC	8.54	2.313	0.00033	0.646
	TC+CC*ApoA1	-7.47E-07	2.83E-07	0.0094	
TBW	TC+CC	7.63	2.269	0.0015	0.671
	TC+CC*ApoB48	-7.35E-05	3.4E-05	0.032	
Weight (TT count: 93; TC+CC count: 178)	TC+CC	15.55	6.514	0.018	0.162
	TC+CC*ghrelin	-0.017	0.011	0.109	
Weight	TC+CC	0.225	5.95	0.0002	0.304
	TC+CC*ApoA1	-2.05E-06	7.64E-07	0.0081	
Weight	TC+CC	9.71	5.41	0.074	0.221
	TC+CC*ApoB48	-5.25	7.98E-05	0.511	

*Results are shown for carrier genotypes regressed against the reference TT genotype. Genetic model was based on dominant mode of inheritance since interactions involving genotypes carrying the effect allele (TC and CC) versus reference genotype (TT) were examined.

### Results From Power Calculation

Results from Power calculation under both “gene only” hypothesis and “gene-environment hypothesis are presented in **Supplementary Table S6**. The power calculation indicated that the study cohort had 80% power to detect associations with the *FTO* variant and could explain 4.1% variance in the traits of SLM, TBW, and weight. The observed effect sizes of 2.249, 1.796, and 3.953 for the association of the *FTO* variant with SLM, TBW, and weight, respectively (see **Table 1**) were less than the expected effect sizes of 2.961, 2.302, and 4.746, respectively, from power calculation under “gene-only” hypothesis; in a similar manner, the observed effect sizes were less than expected effect sizes from power calculation under “gene-environment GxE” hypothesis (see **Supplementary Table S6**).

## Discussion

We showed that the *FTO* variant, rs1421085, was associated with increased body weight, TBW, and SLM in a cohort of Kuwaiti Arab subjects. Further, associations at borderline P-values were observed with lower levels of C-peptide; the inflammatory cytokines, IL-5, TNFα, and IFNγ and the inflammatory chemokine, eotaxin. These observations corroborate the varied associations made by global GWAS between rs1421085 and various physiological processes, including early onset extreme obesity (Wheeler et al., 2013), BMI abnormalities in childhood (Felix et al., 2016), chronotype measurement (morning person) (Jones et al., 2019), percentage body fat (Hubel et al., 2019), lean body mass (fat free mass), type 2 diabetes (Suzuki et al., 2019), energy intake (dietary macronutrient intake) (Tanaka et al., 2013), and hand grip strength (Tikkanen et al., 2018), that were identified in various ethnic groups.

Most notable genetic variants from *FTO* associated with obesity traits are a cluster of as many as 20 common SNPs from its first intron — particularly notable are rs1421085 (the study variant), rs9939609, rs1121980, rs8050136, rs3751812, rs17817449, and rs8050135. The study variant is in strong linkage disequilibrium with all the other above-listed variants (R^2^ > 0.90) except for rs8050135 (R^2^ = 0.0).

In this study, we observed that individuals carrying the rs1421085_C allele exhibited lower levels of inflammatory markers, including IL-4, -5 and -6, TNFα, IFNγ, and MIP1α, among others, while the levels of BMI, TBW, and SLM, as well as leptin and MMP-9, were higher. Associations between *FTO* SNPs and biomarkers show differing data in literature. For example, Zimmermann et al. (2011) found in young Danish men that the *FTO* risk allele, rs9939609, which has a strong linkage disequilibrium with rs1421085, was associated with increased CRP level but had no impact on IL-1, IL-6, and TNFα. However, rs9939609 was associated with increased TNFα in Mexican women with gestational diabetes (Saucedo et al., 2017). The impact of rs9939609 on the fasting and postprandial level of IL-6 and TNFα in Brazilian women revealed that, in the fasting stage, levels of IL-6 and TNFα were similar for the A and T risk alleles but in the postprandial period, while TNFα was not altered in individuals with AA genotype compared to TT and TA genotypes, the serum levels of IL-6 and ghrelin were low and those of leptin were high (Magno et al., 2018). Ethnicity is likely to have an effect on the variation in association of *FTO* with inflammatory markers and investigating these variants may offer a better understanding of their role in the obesity epidemic affecting the world and particularly countries such as Kuwait and others in the Gulf region.

The three body composition traits found associated in this study with the *FTO* variant are linked to one another: SLM is the summation of TBW and protein; LBM is the summation of SLM and minerals; and weight is the sum total of LBM and body fat mass. The genotypes of CC or TC (as compared to the TT genotype) at the *FTO* variant upregulate expression of *FTO* in skeletal muscle (https://gtexportal.org/home/snp/rs1421085), which is a major component of SLM; it is known that an upregulation of *FTO* can result in increased food intake leading to obesity (Church et al., 2010). Similarly, association of *FTO* variants with LBM is known (Livshits et al., 2012). TBW has been observed to be higher in obese women than in non-obese women (Sartorio et al., 2005), and very severely obese individuals have high SLM (along with fat-free mass and waist-hip ratio), followed by severe and moderately obese individuals (Jahanlou and Kouzekanani, 2017).

Apart from the observations that exercise and protein intake increase muscle mass, the main determinants of adult muscle mass are yet to be fully characterised. Family and twin studies (Hsu et al., 2005) have suggested that LBM has a high heritability estimate (range, 0.52–0.60). However, to date, only few genes (*HSD17B11*, *VCAN*, and *ADAMTSL3*) have been linked to lean mass (Zillikens et al., 2017) *albeit* with minor contributions to the trait. The *FTO* gene has been linked to BMI as it not only affects fat tissue and mass, as established in the past, but also relates to muscle mass as described in this study and by others (Zillikens et al., 2017). The report of Antonio et al. (2018) found that the *FTO* variant rs1421085 (subject of our study) was significantly associated with PBF, while our study did not find a significant association. This could be attributed to differences in the cohort studied; the study of Antonio et al. compared non-athletes and athletes, who are likely to show more significant differences in PBF (Antonio et al., 2018).

We showed lower levels of ghrelin, ApoA1, and ApoB48 with the effect allele C at the *FTO* variant in regulating weight, SLM, and TBW. Whereas the association between *FTO* and ghrelin has been reported (Karra et al., 2013) earlier, for the first time, to the best of our knowledge, we report associations between *FTO* and ApoA1 and ApoB48.

Ghrelin, termed the “hunger hormone” and leptin, the “satiety hormone”, alter brain responses to food; high plasma levels of ghrelin and low serum levels of leptin have been linked to the regulation of body weight mediated by *FTO* SNPs (Benedict et al., 2014). ApoA1 is a component of HDL, which is known to transport cholesterol and phospholipids through the bloodstream to the liver. Therefore, our findings were not surprising as *FTO* may interact with ApoA1 in regulating weight, TBW, and SLM and thus affects obesity and also corroborate studies showing SNPs from ApoA1 associated with lower levels of HDL as obesity risk factors (Rashid and Genest, 2007; Chen et al., 2009). In addition, this is also supported by our previous observations showing interactions between Apo-A1/HDL and a genetic variant in *TCN2* regulate WC (Hebbar et al., 2017). Others have demonstrated a positive correlation between ApoB48 and abdominal circumference (Kuo et al., 2019), with obese and hyperlipidemic subjects showing higher levels of ApoB48 (Otokozawa et al., 2009).

SLM contributes to LBM. Low LBM has been shown to be associated with a higher risk of cardiometabolic disorders (Khazem et al., 2018) such as cardiovascular events (Hioki et al., 2015), diabetes (Kalyani et al., 2014), and metabolic syndrome (Kim and Park, 2018). Further, LBM is an important determinant of physical strength, mobility, stamina, and balance (Janssen et al., 2002; Visser et al., 2002; Janssen, 2006) and loss of LBM is associated with sarcopenia (Hansen et al., 1999; Chen et al., 2014; Cruz-Jentoft et al., 2019). It is known that low lean body mass has a strong genetic component, with heritability ranging over 50% (Hsu et al., 2005; Keen-Kim et al., 2006). However, the specific genes underlying the variation in low lean body mass are largely unknown (Urano and Inoue, 2015). Thus, the observation made in our study on FTO variant associated with soft lean mass is novel and important in the field of sarcopenia. Further, physical activity is known to be beneficial in general for the prevention of sarcopenia (Steffl et al., 2017). In this context, it is interesting to note that the studied *FTO* variant has been implicated in affecting BMI *via* gene-by-environment interactions such as overall diet and physical activity, alcohol consumption, and deviations from mean sleep duration (Young et al., 2016; Chang et al., 2018). Thus, the finding that the FTO variant rs1421085 is a determinant of soft lean mass (which is primarily made of skeletal and smooth muscle mass) is important.

The MAF of the rs1421085_T > C variant is 0.23 in 1,000 Genomes Project populations (Genomes Project et al., 2015) and varies considerably across the population with the highest value of 0.43 observed in Europeans and the lowest value of 0.06 observed in Africans. Comparatively, allele frequencies of the variant among individuals in Kuwait and Qatar are 0.42 and 0.49, respectively, similar to that seen in Europeans.

Small sample size can be considered as a limitation of the study; however, it is a novel cohort of Arab participants with deep phenotyping. The SNP frequency is very large (40%) and results of power calculation support the notion that the sample size is adequate to support the association of the *FTO* variant with body weight and lean mass and the interactions with ApoA1, ApoB48, and ghrelin levels.

In conclusion, we report that the *FTO* variant, rs1421085, is a genetic factor for the body composition traits of TBW, SLM, and body weight in Kuwaiti Arabs. Our findings support an association between the *FTO* variant and plasma levels of ghrelin, ApoA1, and ApoB48.

## Data Availability Statement

The raw data supporting the conclusions of this manuscript will be made available by the authors, without undue reservation, to any qualified researcher. Requests for access to data may be addressed to Dr JA at jehad.abubakr@dasmaninstitute.org.

## Ethics Statement

The studies involving human participants were reviewed and approved by Ethical Review Committee of Dasman Diabetes Institute. The patients/participants provided their written informed consent to participate in this study.

## Author Contributions

JA, FA-M, and TAT are responsible for the study design. MA-F and AM performed participants recruitment and collection of samples and phenotype. FA and MM performed genotyping experiments. PH carried out data analysis and contributed to interpreting the results. TAT, MA-F, and PH prepared the manuscript. Manuscript was critically reviewed and approved by FA-M. All authors have read and approved the final manuscript.

## Conflict of Interest

The authors declare that the research was conducted in the absence of any commercial or financial relationships that could be construed as a potential conflict of interest.
