# Supplementary material for: FTO Variant rs1421085 Associates With Increased Body Weight, Soft Lean Mass, and Total Body Water Through Interaction With Ghrelin and Apolipoproteins in Arab Population
Source: Front Genet. 2020 Jan 31;10:1411. doi: 10.3389/fgene.2019.01411 (PMC7006511; doi:10.3389/fgene.2019.01411)
Supplement: Supplementary file 1 [file DataSheet_1.docx]

**Supplementary Tables**

**Supplementary Table S1**: **Demographic characteristics of the study participants, as per genotype distribution at the *FTO* rs1421085 variant, for the considered 60 traits and biomarkers.**

| **Number** | **Traits** | **All (Mean±SD)** | **TT (Mean±SD)** | **TC+CC (Mean±SD)** | **P-value^$^** |
| --- | --- | --- | --- | --- | --- |
| 1 | Sex (Male:Female) | 125:153 | 44:52 | 81:101 | 0.832 |
| 2 | Obesity status (Yes:No) | 134:144 | 41:55 | 93:89 | 0.183 |
| 3 | Diabetes status (Yes:No) | 109:159 | 44:52 | 75:107 | 0.458 |
| 4 | Diabetes medication (Yes:No) | 104:174 | 44:52 | 60:122 | 0.035 |
| 5 | Lipid lowering medication (Yes:No) | 91:187 | 35:61 | 56:126 | 0.336 |
| 6 | Age (years) | 46.25±12.38 | 47.60±12.85 | 45.73±12.06 | 0.241 |
| 7 | Height (m) | 1.64±0.09 | 1.64±0.08 | 1.65±0.09 | 0.257 |
| 8 | Weight (kg) | 81.4±16.23 | 77.70±14.85 | 83.35±16.60 | 0.004 |
| 9 | BMI (kg/m^2^) | 29.9±5.17 | 29.01±5.11 | 30.42±5.18 | 0.032 |
| 10 | WC (cm) | 99.3±13.36 | 97.04±13.52 | 100.65±13.14 | 0.088 |
| 11 | WCHipR | 0.903±0.08 | 0.90±0.087 | 0.90±0.085 | 0.891 |
| 12 | Hip (cm) | 110.4±9.56 | 108.74±8.78 | 111.36±9.88 | 0.074 |
| 13 | SLM (kg) | 48.7±10.13 | 46.48±8.71 | 49.99±10.65 | 0.019 |
| 14 | TBW (litre) | 38.48±7.87 | 36.66±6.75 | 39.46±8.27 | 0.014 |
| 15 | TGL (mmol/l) | 1.22±0.59 | 1.25±0.61 | 1.22±0.59 | 0.649 |
| 16 | HDL (mmol/l) | 1.20±0.32 | 1.22±0.35 | 1.18±0.31 | 0.357 |
| 17 | LDL (mmol/l) | 3.13±0.96 | 3.13±0.98 | 3.14±0.96 | 0.961 |
| 18 | TC (mmol/l) | 5.02±1.09 | 5.01±1.12 | 5.04±1.10 | 0.902 |
| 19 | FPG (mmol/l) | 5.77±1.24 | 5.87±1.37 | 5.73±1.17 | 0.413 |
| 20 | HbA1C (%) | 6.31±1.29 | 6.55±1.43 | 6.19±1.21 | 0.050 |
| 21 | Insulin (pg/ml) | 3.53±1.53 | 3.38±1.43 | 3.63±1.58 | 0.295 |
| 22 | HsCRP (mg/ml) | 4.23±3.34 | 4.20±3.37 | 4.25±3.35 | 0.942 |
| 23 | Eotaxin (ng/ml) | 69.74±20.49 | 75.92±22.86 | 66.76±18.63 | 0.012 |
| 24 | PBF (%) | 35.5±5.68 | 35.07±5.53 | 35.76±5.76 | 0.437 |
| 25 | WBC | 6.85±1.90 | 6.98±1.75 | 6.78±1.98 | 0.408 |
| 26 | IFNγ (pg/ml) | 165.24±23.02 | 181.03±57.89 | 157.05±46.0 | 0.007 |
| 27 | IL1ra (pg/ml) | 219.7±157.6 | 190.12±124.2 | 234.29±170.7 | 0.070 |
| 28 | IL1b (pg/ml) | 8.46±2.60 | 9.19±2.99 | 8.1±2.33 | 0.019 |
| 29 | IL4 (pg/ml) | 6.18±1.72 | 6.66±2.06 | 5.94±1.47 | 0.020 |
| 30 | IL5 (pg/ml) | 18.68±8.4 | 21.86±8.86 | 17.06±7.74 | 0.0007 |
| 31 | IL6 (pg/ml) | 16.96±4.7 | 18.53±5.57 | 16.13±4.0 | 0.0046 |
| 32 | IL7 (pg/ml) | 13.23±5.75 | 15.18±6.25 | 12.25±5.28 | 0.0004 |
| 33 | IL8 (pg/ml) | 41.33±14.5 | 44.85±15.62 | 39.48±13.66 | 0.032 |
| 34 | IL9 (pg/ml) | 73.14±41.4 | 82.41±40.73 | 68.11±41.24 | 0.032 |
| 35 | IL10 (pg/ml) | 26.16±13.8 | 29.64±16.56 | 24.47±12.02 | 0.047 |
| 36 | IL13 (pg/ml) | 9.67±4.84 | 9.74±4.53 | 9.57±4.99 | 0.827 |
| 37 | IL17 (pg/ml) | 72.15±40.9 | 80.62±45.76 | 67.69±37.97 | 0.082 |
| 38 | IP10 (pg/ml) | 466.42±160.8 | 479.45±165.9 | 459.83±159.2 | 0.478 |
| 39 | MIP1α (pg/ml) | 9.35±3.61 | 10.35±4.23 | 8.83±3.14 | 0.018 |
| 40 | C-peptide (pg/ml) | 1446.8±780.1 | 1341.29±776.3 | 1484.91±758.3 | 0.260 |
| 41 | GCSF (pg/ml) | 63.45±23.02 | 68.36±25.62 | 60.77±21.26 | 0.061 |
| 42 | TNFα (ng/ml) | 127.67±32.16 | 137.66±33.19 | 122.51±30.6 | 0.005 |
| 43 | Leptin (pg/ml) | 7193.3±4138.2 | 6321.3±3878 | 7687.68±4230 | 0.037 |
| 44 | MMP9 (pg/ml) | 7649.5±4132.9 | 6793.08±3183 | 8072.75±4496 | 0.022 |
| 45 | ApoA1_A (pg/ml) | 1600955±429737 | 1551513.4±388443 | 1632017.5±454411 | 0.197 |
| 46 | ApoA1 (ng/ml) | 7044393±2787263 | 7472685.42±3057692 | 6824887.6±2644611 | 0.161 |
| 47 | ApoB48 (ng/ml) | 60632.7±27687.9 | 63371.85±26851.3 | 59038.51±28356.3 | 0.298 |
| 48 | Apo B (ng/ml) | 1829168±774965.6 | 1805424.1±729027 | 1850537.5±804346 | 0.683 |
| 49 | Apo A2 (ng/ml) | 1001467±426735.3 | 1004701.8±428048 | 1004940.6±426600 | 0.996 |
| 50 | Apo C1 (ng/ml) | 477763.6±109961.6 | 485590.2±98371.1 | 475280.7±115577 | 0.531 |
| 51 | Apo C3 (ng/ml) | 296191.9±164658 | 282191.4±156204.7 | 305608.5±169691 | 0.314 |
| 52 | Apo D (ng/ml) | 62272.9±29231.5 | 62374.94±30482.96 | 62509.49±28819.37 | 0.975 |
| 53 | Apo E (ng/ml) | 97171.6±47594 | 94472.11±45649.9 | 99095.5±48535.6 | 0.502 |
| 54 | Apo H (ng/ml) | 1380141±537713 | 1312601.7±512008 | 1421889.8±548231 | 0.160 |
| 55 | Apo J (ng/ml) | 271594.1±110527.6 | 262693.7±103049 | 277925.16±114378 | 0.336 |
| 56 | RANTES (pg/ml) | 58742.58±33796.3 | 7933.47±1685.3 | 7849.4±1482.1 | 0.747 |
| 57 | RAGE (pg/ml) | 102.6±59.06 | 113.46±60.09 | 97.63±58.33 | 0.068 |
| 58 | GLP1 (pg/ml) | 271.3±44.5 | 275.18±47.2 | 268.67±42.81 | 0.390 |
| 59 | Glucagon (pg/ml) | 158.4±47.28 | 160.76±49.36 | 156.91±46.47 | 0.625 |
| 60 | Adiponectin | 4294.1±2497.6 | 4346.07±2632.9 | 4258.3±2455.2 | 0.817 |
| 61 | Adipsin (ng/ml) | 5139.7±1875.9 | 5215.5±1736.4 | 5114.67±1966.3 | 0.736 |
| 62 | Vitamin-D (ng/ml) | 26.43±15.9 | 28.26±17.57 | 25.52±14.9 | 0.329 |
| 63 | Vesfatin (pg/ml) | 3681.9±1154.1 | 3757.6±1165.4 | 3634.6±1154.3 | 0.526 |
| 64 | MIP1α (pg/ml) | 263.58±98.1 | 267.79±110.06 | 261.36±92.1 | 0.706 |
| 65 | PAI1 (pg/ml) | 14653.3±4887.8 | 14365.72±4946.3 | 14674.8±4724.6 | 0.702 |
| 66 | Ghrelin (pg/ml) | 546.9±215.7 | 569.7±233.5 | 536.94±205.7 | 0.379 |

**^$^, Chi-square test was used for categorical variables and Student’s t-test was used for quantitative variables.**

**Supplementary Table S2:** **Results of association tests, using genetic model based on additive mode of inheritance, for the *FTO* variant (with C as the risk allele) with the considered phenotype traits.** Association tests were adjusted for age, sex (Regular correction) and for the further confounders of diabetes medication (DM) and lipid lowering medication (LLM). Associations showing P-value ≤ 0.05 were seen with 15 traits (including the SLM, TBW and weight traits listed in Table 2); in a further case of 3 traits (namely hip, leptin, and WC), the associations reached the threshold when the tests were corrected for medication. Except for the traits of weight, SLM and TBW, none reached a P_emp_-Value ≤ 0.05.

| **Trait** | **Sample Size** | **Effect size (Beta value)** | **P-value** | **P*_emp_*-value** |
| --- | --- | --- | --- | --- |
| BMI | R | 0.9091 | 0.04351 | 0.892 |
|  | DM | 1.121 | 0.01108 | 0.4352 |
|  | LLM | 1.068 | 0.01553 | 0.5534 |
| C-peptide | R | -0.5298 | 0.004394 | 0.206 |
|  | R+DM | -0.5631 | 0.002827 | 0.1387 |
|  | R+LLM | -0.5561 | 0.003256 | 0.1538 |
| Height | R | 0.01176 | 0.02888 | 0.774 |
|  | R+DM | 0.01175 | 0.0305 | 0.7958 |
|  | R+LLM | 0.0117 | 0.03083 | 0.803 |
| Eotaxin | R | -6.081 | 0.007896 | 0.3368 |
|  | R+DM | -5.493 | 0.01584 | 0.5502 |
|  | R+LLM | -5.704 | 0.0126 | 0.4806 |
| GCSF | R | -5.372 | 0.04131 | 0.881 |
|  | R+DM | -5.092 | 0.05577 | 0.9418 |
|  | R+LLM | -5.078 | 0.05558 | 0.9402 |
| IFNγ | R | -15.84 | 0.005743 | 0.252 |
|  | R+DM | -15.29 | 0.008169 | 0.3438 |
|  | R+LLM | -15.3 | 0.008124 | 0.337 |
| IL1ra | R | 37.88 | 0.03817 | 0.8617 |
|  | R+DM | 39.75 | 0.03087 | 0.7876 |
|  | R+LLM | 39.35 | 0.03331 | 0.8086 |
| IL5 | R | -2.491 | 0.008431 | 0.3602 |
|  | R+DM | -2.304 | 0.01541 | 0.5498 |
|  | R+LLM | -2.439 | 0.01065 | 0.4253 |
| IL6 | R | -1.381 | 0.01041 | 0.4179 |
|  | R+DM | -1.347 | 0.01296 | 0.4781 |
|  | R+LLM | -1.362 | 0.01224 | 0.4704 |
| IL7 | R | -1.509 | 0.02031 | 0.6499 |
|  | R+DM | -1.331 | 0.04041 | 0.8734 |
|  | R+LLM | -1.372 | 0.03665 | 0.8525 |
| MIP1α | R | -0.9093 | 0.02788 | 0.7592 |
|  | R+DM | -0.8852 | 0.03351 | 0.822 |
|  | R+LLM | -0.885 | 0.03351 | 0.8207 |
| TNFα | R | -9.854 | 0.006885 | 0.2912 |
|  | R+DM | -9.258 | 0.0113 | 0.4396 |
|  | R+LLM | -9.305 | 0.01093 | 0.4284 |
| SLM | R | 2.249 | 0.001029 | 0.05539 |
|  | DM | 2.402 | 0.0003009 | 0.0175 |
|  | LLM | 2.265 | 0.0006685 | 0.0331 |
| TBW | R | 1.796 | 0.001031 | 0.05579 |
|  | R+DM | 1.92 | 0.0002925 | 0.0161 |
|  | R+LLM | 1.808 | 0.0006618 | 0.037 |
| Weight | R | 3.953 | 0.00221 | 0.1144 |
|  | DM | 4.566 | 0.0003021 | 0.0183 |
|  | LLM | 4.374 | 0.0005456 | 0.0307 |
| **Instances wherein the associations reach statistical significance (≤ 0.05) when corrections were made for medication.** | | | | |
| Hip | R | 1.804 | 0.08326 | 0.9876 |
|  | R+DM | 2.073 | 0.04462 | 0.9029 |
|  | R+LLM | 1.832 | 0.07327 | 0.9768 |
| Waist circumference | R | 2.424 | 0.06121 | 0.9618 |
|  | R+DM | 2.963 | 0.01483 | 0.5262 |
|  | R+LLM | 2.525 | 0.03909 | 0.87 |
| Leptin | R | 729.9 | 0.05717 | 0.9505 |
|  | R+DM | 846 | 0.02708 | 0.7535 |
|  | R+LLM | 822.4 | 0.03127 | 0.8125 |

**Supplementary Table S3.** Differences in the mean values of weight, SLM and TBW between the genotype distributions of difference between TC+CC and TT among non-obese, obese, non-diabetic and diabetic cohorts**.**

| Trait | Cohort | TC+CC (Mean±SD) | TT (Mean±SD) | P-value |
| --- | --- | --- | --- | --- |
| Weight | Non-obese | 71.802±11.79 | 68.342 ± 10.94 | 0.0806 |
|  | Obese | 94.395 ± 12.51 | 90.103 ± 9.13 | **0.0301** |
|  | Non-diabetic | 79.864 ± 16.84 | 73.384 ± 15.19 | **0.0193** |
|  | Diabetic | 88.254 ± 15.03 | 82.509 ± 12.99 | **0.0309** |
|  | Non-hypertensive | 74.25 ± 15.70 | 81.45 ± 17.01 | **0.005** |
|  | Hypertensive | 83.97 ± 10.76 | 88.40 ± 14.56 | 0.115 |
|  |  |  |  |  |
| SLM | Non-obese | 45.17 ± 9.20 | 43.19 ± 7.58 | 0.280 |
|  | Obese | 53.77 ± 10.22 | 50.75 ± 8.32 | 0.144 |
|  | Non-diabetic | 48.10 ± 10.72 | 43.75 ± 8.61 | **0.039** |
|  | Diabetic | 52.23 ± 10.21 | 49.05 ± 8.11 | 0.116 |
|  | Non-hypertensive | 48.93 ± 10.24 | 44.68 ± 8.46 | **0.022** |
|  | Hypertensive | 52.15 ± 11.27 | 48.98 ± 8.59 | 0.207 |
|  |  |  |  |  |
| TBW | Non-obese | 35.49 ± 7.07 | 33.97 ± 5.85 | 0.281 |
|  | Obese | 42.57 ± 7.85 | 40.15 ± 6.31 | 0.125 |
|  | Non-diabetic | 37.94 ± 8.34 | 34.48 ± 6.68 | **0.035** |
|  | Diabetic | 41.26 ± 7.89 | 38.71 ± 6.25 | 0.103 |
|  | Non-hypertensive | 38.62 ± 8.00 | 35.21 ± 6.57 | **0.018** |
|  | Hypertensive | 41.17 ± 8.67 | 38.67 ± 6.59 | 0.196 |

**Supplementary Table S4. Linear regression model between the genotypes containing the effect allele and the traits of SLM, TBW and weight with “obese status” and “diabetes status” of the participant as an interaction term.**

| **Trait (response variable)** | **Genotype and/or interacting trait (Predict variable)** | **Estimate** | **Std. Error** | **P-value** | **Adjusted R-square (model)** |
| --- | --- | --- | --- | --- | --- |
| SLM | TC+CC | -1.237 | 2.522 | 0.626 | 0.747 |
|  | TC+CC*Obese status | 2.391 | 1.617 | 0.141 |  |
| SLM | TC+CC | 1.590 | 3.048 | 0.603 | 0.636 |
|  | TC+CC*Diabetes status | 1.197 | 1.936 | 0.537 |  |
| TBW | TC+CC | -1.036 | 1.973 | 0.600 | 0.744 |
|  | TC+CC*Obese status | 1.933 | 1.265 | 0.128 |  |
| TBW | TC+CC | 1.380 | 2.431 | 0.571 | 0.617 |
|  | TC+CC*Diabetes Status | 0.892 | 1.544 | 0.564 |  |
| Weight | TC+CC | 2.905 | 3.754 | 0.440 | 0.659 |
|  | TC+CC*Obese status | 0.967 | 2.437 | 0.692 |  |
| Weight | TC+CC | 5.454 | 5.576 | 0.328 | 0.245 |
|  | TC+CC*Diabetes Status | 0.778 | 3.633 | 0.831 |  |

**Supplementary Table S5: Correlations between body weight, SLM and TBW with the other traits and biomarkers.** Pearson correlation analysis was performed. Values of correlation coefficients ≥ 0.20 are shown in green font.

| **Number** | **Traits** | **Correlation with weight** | **P-values of correlation with weight** | **Correlation with SLM** | **P-value of correlation with SLM** | **Correlation with TBW** | **P-value of correlation with TBW** |
| --- | --- | --- | --- | --- | --- | --- | --- |
| 1 | Height (m) | 0.56 | 4.20E-24 | 0.83 | 2.60E-45 | 0.84 | 1.50E-47 |
| 2 | Weight (kg) |  |  | 0.89 | 2.40E-62 | 0.88 | 2.60E-57 |
| 3 | BMI (kg/m^2^) | 0.82 | 3.70E-69 | 0.51 | 2.80E-13 | 0.48 | 7.50E-12 |
| 4 | WC (cm) | 0.85 | 5.70E-50 | 0.7 | 1.10E-26 | 0.68 | 6.90E-25 |
| 5 | WCHipR | 0.53 | 6.40E-14 | 0.6 | 4.00E-18 | 0.6 | 3.80E-18 |
| 6 | Hip (cm) | 0.68 | 9.40E-25 | 0.38 | 2.50E-07 | 0.36 | 1.90E-06 |
| 7 | SLM (kg) | 0.88 | 2.60E-57 | 1 | 5.30E-258 |  |  |
| 8 | TBW (litre) | 0.89 | 2.40E-62 |  |  | 1 | 5.30E-258 |
| 9 | TGL (mmol/l) | 0.33 | 9.70E-08 | 0.34 | 5.50E-06 | 0.34 | 5.20E-06 |
| 10 | HDL (mmol/l) | -0.3 | 1.10E-06 | -0.45 | 1.00E-09 | -0.45 | 9.30E-10 |
| 11 | LDL (mmol/l) | -0.01 | 8.70E-01 | -0.01 | 9.40E-01 | 0 | 9.50E-01 |
| 12 | TC (mmol/l) | -0.09 | 1.40E-01 | -0.11 | 1.40E-01 | -0.11 | 1.40E-01 |
| 13 | FPG (mmol/l) | 0.25 | 8.80E-05 | 0.27 | 7.30E-04 | 0.27 | 8.00E-04 |
| 14 | HbA1C (%) | 0.3 | 1.60E-06 | 0.2 | 1.30E-02 | 0.19 | 1.70E-02 |
| 15 | Insulin (pg/ml) | 0.25 | 5.90E-04 | 0.12 | 1.80E-01 | 0.11 | 1.90E-01 |
| 16 | HsCRP (mg/ml) | 0.17 | 1.10E-01 | -0.02 | 8.90E-01 | -0.03 | 8.20E-01 |
| 17 | Eotaxin (ng/ml) | -0.04 | 6.30E-01 | 0.04 | 6.40E-01 | 0.05 | 6.20E-01 |
| 18 | PBF (%) | 0.14 | 6.20E-02 | -0.31 | 2.60E-05 | -0.34 | 3.10E-06 |
| 19 | WBC | 0.22 | 3.80E-04 | 0.16 | 3.50E-02 | 0.15 | 4.20E-02 |
| 20 | IFNg (pg/ml) | -0.15 | 5.00E-02 | -0.13 | 1.70E-01 | -0.13 | 1.80E-01 |
| 21 | IL1ra (pg/ml) | -0.03 | 7.10E-01 | -0.07 | 5.10E-01 | -0.07 | 5.10E-01 |
| 22 | IL1b (pg/ml) | -0.17 | 2.70E-02 | -0.12 | 2.00E-01 | -0.11 | 2.30E-01 |
| 23 | IL4 (pg/ml) | -0.12 | 1.10E-01 | -0.09 | 3.60E-01 | -0.08 | 3.80E-01 |
| 24 | IL5 (pg/ml) | -0.08 | 3.10E-01 | 0.04 | 6.50E-01 | 0.05 | 6.20E-01 |
| 25 | IL6 (pg/ml) | 0 | 9.70E-01 | 0.01 | 9.40E-01 | 0.01 | 9.10E-01 |
| 26 | IL7 (pg/ml) | 0.05 | 5.60E-01 | 0.14 | 1.50E-01 | 0.14 | 1.40E-01 |
| 27 | IL8 (pg/ml) | -0.12 | 1.40E-01 | -0.04 | 6.50E-01 | -0.04 | 6.80E-01 |
| 28 | IL9 (pg/ml) | 0.03 | 7.40E-01 | -0.01 | 8.80E-01 | -0.01 | 8.80E-01 |
| 29 | IL10 (pg/ml) | -0.03 | 6.80E-01 | 0 | 9.60E-01 | 0.01 | 9.30E-01 |
| 30 | IL13 (pg/ml) | 0.14 | 7.50E-02 | 0.16 | 1.10E-01 | 0.16 | 9.70E-02 |
| 31 | IL17 (pg/ml) | -0.12 | 1.30E-01 | 0 | 1.00E+00 | 0 | 1.00E+00 |
| 32 | IP10 (pg/ml) | -0.1 | 2.30E-01 | -0.08 | 4.20E-01 | -0.09 | 3.90E-01 |
| 33 | MIP1a (pg/ml) | -0.17 | 3.20E-02 | -0.18 | 5.20E-02 | -0.18 | 6.20E-02 |
| 34 | C-peptide (pg/ml) | -0.03 | 7.40E-01 | -0.06 | 5.00E-01 | -0.06 | 5.10E-01 |
| 35 | GCSF (pg/ml) | -0.1 | 2.20E-01 | 0 | 9.90E-01 | 0.01 | 9.50E-01 |
| 36 | TNFa (ng/ml) | -0.16 | 4.60E-02 | -0.13 | 1.80E-01 | -0.12 | 2.00E-01 |
| 37 | Leptin (pg/ml) | 0.13 | 8.90E-02 | -0.22 | 2.20E-02 | -0.24 | 1.10E-02 |
| 38 | MMP9 (pg/ml) | -0.01 | 9.30E-01 | 0.11 | 2.40E-01 | 0.11 | 2.20E-01 |
| 39 | Apo A1_A (pg/ml) | -0.01 | 9.40E-01 | -0.02 | 8.60E-01 | -0.02 | 8.50E-01 |
| 40 | Apo A1 (ng/ml) | -0.05 | 5.20E-01 | -0.06 | 4.60E-01 | -0.06 | 4.70E-01 |
| 41 | Apo B48 (ng/ml) | **-0.25** | 6.30E-04 | **-0.23** | 1.30E-02 | -0.22 | 1.60E-02 |
| 42 | Apo B (ng/ml) | 0.09 | 2.00E-01 | 0.11 | 2.10E-01 | 0.11 | 2.20E-01 |
| 43 | Apo A2 (ng/ml) | 0.02 | 8.00E-01 | 0.02 | 8.00E-01 | 0.02 | 8.10E-01 |
| 44 | Apo C1 (ng/ml) | -0.07 | 3.30E-01 | -0.08 | 3.80E-01 | -0.08 | 3.80E-01 |
| 45 | Apo C3 (ng/ml) | 0.22 | 1.20E-03 | 0.15 | 8.10E-02 | 0.15 | 9.10E-02 |
| 46 | Apo D (ng/ml) | -0.01 | 8.50E-01 | 0.01 | 8.70E-01 | 0.02 | 8.50E-01 |
| 47 | Apo E (ng/ml) | -0.01 | 9.10E-01 | -0.05 | 5.70E-01 | -0.05 | 5.30E-01 |
| 48 | Apo H (ng/ml) | 0.08 | 2.60E-01 | 0.15 | 8.30E-02 | 0.16 | 8.00E-02 |
| 49 | Apo J (ng/ml) | 0.1 | 1.70E-01 | 0.04 | 6.30E-01 | 0.04 | 6.40E-01 |
| 50 | RANTES (pg/ml) | 0.09 | 2.10E-01 | 0.08 | 3.80E-01 | 0.08 | 3.70E-01 |
| 51 | RAGE (pg/ml) | -0.13 | 5.60E-02 | -0.08 | 3.20E-01 | -0.08 | 3.50E-01 |
| 52 | GLP1 (pg/ml) | 0.18 | 2.50E-02 | 0.07 | 4.90E-01 | 0.07 | 4.70E-01 |
| 53 | Glucagon (pg/ml) | 0.26 | 8.70E-04 | 0.32 | 4.80E-04 | 0.32 | 4.70E-04 |
| 54 | Adiponectin | -0.32 | 2.20E-06 | -0.37 | 4.90E-06 | -0.38 | 4.60E-06 |
| 55 | Adipsin (ng/ml) | 0.2 | 9.80E-03 | 0.28 | 1.50E-03 | 0.28 | 1.50E-03 |
| 56 | Vitamin-D (ng/ml) | -0.14 | 8.60E-02 | -0.09 | 4.00E-01 | -0.08 | 4.40E-01 |
| 57 | Vesfatin (pg/ml) | 0.07 | 4.10E-01 | 0.02 | 8.20E-01 | 0.03 | 7.90E-01 |
| 58 | MIP1a (pg/ml) | -0.17 | 3.20E-02 | -0.18 | 5.20E-02 | -0.18 | 6.20E-02 |
| 59 | PAI1 (pg/ml) | 0.07 | 3.50E-01 | 0.09 | 3.70E-01 | 0.08 | 3.80E-01 |
| 60 | Ghrelin (pg/ml) | -0.18 | 2.40E-02 | **-0.27** | 4.30E-03 | -0.27 | 4.80E-03 |

**Supplementary Table S6. Power Calculation for the association of the *FTO* rs1421085_C variant (MAF=40%) with SLM, TBW and weight traits. Calculations for “gene only” hypothesis were performed using additive genetic model (TT versus TC versus CC); calculations for “gene-environment GxE” hypothesis were performed using dominant genetic model (TT versus (TC+CC)).**

| **Marginal Rsq** | **Sample Size** | **Expected effect size considering “Gene only” hypothesis (response variable)** | **Expected effect size considering GXE hypothesis (E=Ghrelin)** | **Expected effect size considering GXE hypothesis (E=ApoA1)** | **Expected effect size considering GXE hypothesis (E=ApoB48)** |
| --- | --- | --- | --- | --- | --- |
| **Power Calculation for the trait of SLM** | | | | | |
| 0.0010 | 7845 | 0.4624 | 0.0021 | 0.0000 | 0.0000 |
| 0.0060 | 1304 | 1.1326 | 0.0052 | 0.0000 | 0.0000 |
| 0.0110 | 710 | 1.5335 | 0.0070 | 0.0000 | 0.0000 |
| 0.0160 | 487 | 1.8495 | 0.0085 | 0.0000 | 0.0001 |
| 0.0210 | 370 | 2.1188 | 0.0097 | 0.0000 | 0.0001 |
| 0.0260 | 298 | 2.3576 | 0.0108 | 0.0000 | 0.0001 |
| 0.0310 | 249 | 2.5744 | 0.0118 | 0.0000 | 0.0001 |
| 0.0360 | 214 | 2.7742 | 0.0127 | 0.0000 | 0.0001 |
| **0.0410** | **187** | **2.9606** | **0.0135** | **0.0000** | 0.0001 |
| 0.0460 | 167 | 3.1359 | 0.0144 | 0.0000 | 0.0001 |
| **Power Calculation for the trait of TBW** | | | | | |
| 0.0010 | 7845 | 0.3595 | 0.0017 | 0.0000 | 0.0000 |
| 0.0060 | 1304 | 0.8806 | 0.0041 | 0.0000 | 0.0000 |
| 0.0110 | 710 | 1.1923 | 0.0055 | 0.0000 | 0.0000 |
| 0.0160 | 487 | 1.4380 | 0.0067 | 0.0000 | 0.0001 |
| 0.0210 | 370 | 1.6474 | 0.0076 | 0.0000 | 0.0001 |
| 0.0260 | 298 | 1.8330 | 0.0085 | 0.0000 | 0.0001 |
| 0.0310 | 249 | 2.0015 | 0.0093 | 0.0000 | 0.0001 |
| 0.0360 | 214 | 2.1569 | 0.0100 | 0.0000 | 0.0001 |
| **0.0410** | **187** | **2.3018** | **0.0107** | 0.0000 | **0.0001** |
| 0.0460 | 167 | 2.4382 | 0.0013 | 0.0000 | 0.0001 |
| **Power Calculation for the trait of weight** | | | | | |
| 0.0010 | 7845 | 0.7413 | 0.0034 | 0.0000 | 0.0000 |
| 0.0060 | 1304 | 1.8157 | 0.0084 | 0.0000 | 0.0001 |
| 0.0110 | 710 | 2.4585 | 0.0114 | 0.0000 | 0.0001 |
| 0.0160 | 487 | 2.9650 | 0.0137 | 0.0000 | 0.0001 |
| 0.0210 | 370 | 3.3968 | 0.0157 | 0.0000 | 0.0001 |
| 0.0260 | 298 | 3.7797 | 0.0175 | 0.0000 | 0.0001 |
| 0.0310 | 249 | 4.1271 | 0.0191 | 0.0000 | 0.0001 |
| 0.0360 | 214 | 4.4475 | 0.0206 | 0.0000 | 0.0002 |
| **0.0410** | **187** | **4.7463** | **0.0220** | 0.0000 | 0.0002 |
| 0.0460 | 167 | 5.0274 | 0.0233 | 0.0000 | 0.0002 |
